# Prevalence of Patellofemoral Pain and Knee Pain in the General Population of Saudi Arabia

**DOI:** 10.7759/cureus.30355

**Published:** 2022-10-16

**Authors:** Sarah S Aldharman, Haneen H Almuhammadi, Abdullah Y Madkhali, Raad A Alnami, Mohammed A Alkadi, Danah M Albalawi, Yousef A Alhamaid, Zenat A Khired

**Affiliations:** 1 Medicine, King Saud Bin Abdulaziz University for Health Sciences, Riyadh, SAU; 2 Medicine, Taibah University, Madina, SAU; 3 Medicine, Jazan University, Jazan, SAU; 4 Medicine and Surgery, King Khalid University, Abha, SAU; 5 Medicine, Najran University, Najran, SAU; 6 Medicine, University of Tabuk, Tabuk, SAU; 7 Medicine, King Faisal University, Alahsa, SAU; 8 Surgery, Jazan University, Jazan, SAU

**Keywords:** saudi arabia, patellofemoral, prevalence, knee pain, general population

## Abstract

Background

Patellofemoral pain (PFP) is a common cause of knee pain. This condition can restrict daily activities by trying to avoid activities that aggravate their pain. This study aimed to determine the prevalence of PFP and knee pain and its associated factors among Saudi young adults.

Methods

A descriptive cross-sectional study was conducted in Saudi Arabia. A validated translated Arabic questionnaire was used. Data was collected through an online self-administered questionnaire. Saudi young adults of both genders aged between (18 to 40 years) were included. The mean ±SD was described for continuous variables, whereas categorical variables were reported using frequencies and percentages. The chi-square test was used for descriptive analysis.

Results

A total of 1558 subjects were enrolled in the current study. About 663 (42.6%) were males and 895 (57.4%) were females. Of the total participants, 718 (46%) were within the age group of 18 to 25. The overall prevalence of PFP among the current study participant was found to be 30.3%. The prevalence of PFP among males was found to be 31.4% and the prevalence of PFP among females was found to be 29.5%. The overall prevalence of knee pain among study participants was found to be 13.2% as 205 of the participants reported knee pain. The prevalence of knee pain among males was found to be 14% and the prevalence of knee pain among females was found to be 12.3%. The multivariate analysis included the following variables: age, gender, and marital status. The following factors predicted higher rate of PFP: being 18 to 25 years old (p-value < 0.001, odds ratio = 1), being 26 to 35 years old (p-value = 0.001, odds ratio = 1.689).

Conclusion

The prevalence of PFP and knee pains was found to be relatively high in Saudi Arabia. Age less than 40 years old was found to be associated with a higher prevalence of PFP and knee pain when compared to other age groups.

## Introduction

The knee is a very complex structure, and it is considered one of the largest joints in our body [1]. Patellofemoral pain syndrome (PFPS) is one of the most common causes of knee pain [2]. It can be defined as pain behind the patella that presents on a flexed knee while performing specific activities that put a weight-bearing load on the patellofemoral joint [3]. It is usually more prevalent in females, and it also tends to affect adolescents, athletes, and active adults [3,4]. Unfortunately, this condition can restrict their daily living by trying to avoid activities that aggravate their pain [5]. At the same time, 40% to 57% of patients do not exhibit favorable long-term outcomes [6].

A systematic review in 2018 mentioned that patellofemoral pain is one of the most frequent types of knee discomfort [4]. There are variations in reported incidence and prevalence, and there is a clear need to adequately describe the epidemiology of patellofemoral pain before allocating healthcare and research funding. Patellofemoral pain was found to be prevalent in 22.7% of the general population and 28.9% of adolescents [4]. Given this, as well as the poor long-term prognosis and high levels of disability, PFP should be a key focus of a study [4]. Another key aspect of PFP is psychological health, which was highlighted by a study conducted in the UK that aimed to assess the prevalence of anxiety and depression among PFP patients [7]. Results showed that half of the respondents (49.5 %) experienced anxiety, while 20.8% experienced depression. People with PFP have higher rates of anxiety and depression than the general population [7]. These findings highlight the need for more research into the implications of psychological aspects of PFP, such as anxiety and sadness.

In 2018, a study in China was carried out to assess the prevalence of PFP and knee pain in a general population of young adults and to investigate if gender, age, or body mass index (BMI) were linked to PFP [5]. Patellofemoral pain was found to be present in 20.7 % of participants, and knee discomfort was found to be prevalent in 35.6 % of participants. Gender, age, and BMI were not found to be significant predictors of PFP prevalence [5]. A cross-sectional study was conducted in Denmark to evaluate the prevalence of pain medication use for adolescent knee discomfort, as well as the factors that influence use [8]. The use of pain medications for knee pain by adolescents was reported by 21%, and knee-related symptoms were the most consistently linked factor to the usage of pain medications [8]. A Saudi study looked at the prevalence of PFP in Majmaah, Saudi Arabia [9]. Females had a higher prevalence of PFP (72.3%), whereas males had a lower prevalence of (27.7%). Furthermore, age was a significant predictor of PFP, while neither gender nor BMI was a significant predictor [9].

Due to the complex nature of PFP, pinpointing the exact source of pain in a single patient can be challenging. Understanding how demographic heterogeneity affects these numerous parameters would help researchers reach a better consensus on the cause of PFP. Thus, a retrospective study investigated the hypothesis that sex, height, weight, BMI, and age influence patellofemoral kinematics [10]. Weight and BMI had a significant effect on the patellar shift [10].

The bulk of investigations on the prevalence of PFP has been confined to military people, sports groups, or school students. Little research has looked at data from general populations. A previous local study conducted in Saudi Arabia focused on the prevalence of PFP in a specific region [9]. However, there is no regional study investigating this issue on a larger scale in different regions of Saudi Arabia. Therefore, the current study aimed to determine the prevalence of PFP and knee pain in the general population of Saudi young adults and to identify potential associated factors. We believe that studying the epidemiology of this topic can significantly help in understanding the etiology and risk factors in our population, which can help in preventing this condition.

## Materials and methods

A descriptive cross-sectional questionnaire-based study was conducted in Saudi Arabia between May 2022 and June 2022. The target population was the general population of Saudi young adults from different regions (Central, Southern, Eastern, Western, and Northern). The data was gathered via a self-administered questionnaire. The questionnaire was distributed electronically via Google Forms (Google LLC, Mountain View, CA, USA). The data were entered into Microsoft Excel (Microsoft Corp., Redmond, WA, USA) and then uploaded and analyzed using the Statistical Package for the Social Sciences (SPSS) software (IBM Corp., Armonk, NY, USA).

Inclusion criteria and exclusion criteria

The general population of Saudi young adults of both genders aged between 18 to 40 years was included in this study. Participants who were outside the age range or did not fill out the whole questionnaire were excluded from the study.

Sampling technique and sample size calculation

OpenEpi® version 3.0 software was employed to estimate our sample size which is representative of Saudi Arabia's population of 34 million. The representative sample size needed is 385, with a margin error of 5% and a confidence level of 95%. We intended to get more than the estimated sample size to account for any exclusions. Non-probability consecutive sampling technique was used.

Data collection instrument and procedures

The data was collected through a self-report questionnaire, the Survey instrument for Natural history, Aetiology and Prevalence of Patellofemoral pain Studies (SNAPPS) developed by Dey et al. in 2016 [11]. The Arabic version of the questionnaire on their website was used to make it easier for the public to read and understand [12]. The questionnaire was created to distinguish between those in the community who had PFP and those who did not. 

The SNAPPS questionnaire has four sections. The first section included demographic information and identified people who had knee pain based on a single question. The second section covered the clinical characteristics of the knee issue. The third component dealt with the difficulty or pain experienced when performing specific tasks. The fourth section was meant to pinpoint the location of the pain using a knee pain map. 

If the participant does not have knee pain or problems based on the answer in the first section, the questionnaire would be submitted, and the participant would be placed in the category of patients with no knee pain. Participants who do have knee pain and answered "yes" to the first section would continue to the remaining sections and their total score was given by the sum of the scores from sections two and four. The third section is currently not scored because the results showed that both sections two and four had high sensitivity and specificity (>90%) and had satisfactory measurement properties based on the scoring. Therefore, our online SNAPPS questionnaire included sections one, two, and four from the original questionnaire. Section two consisted of seven questions, and participants may receive a score of "0" or "1" for each question based on their response. Section four had an image of a knee joint with labels identifying the medial, lateral, and inferior portions of the patella. Both knees had a total of six locations that were identified. The participants were instructed to identify the number of places on both knees where they felt pain. Each specified pain location received one point. The minimum score of the questionnaire was 0 and the maximum score was 13. Participants with a total score <6 were considered to have self-reported knee pain but not PFP. With a total score of 6 or above, the participants were considered to have patellofemoral pain.

An electronic Google form survey was distributed on several social media platforms including WhatsApp, Twitter, and Telegram. Using Google Forms features such as “required to proceed” to make sure the study criteria would be fulfilled, a question was provided at the beginning of the questionnaire " Is your age between 18-40?". If the answer was "yes", the participant would continue to go through questions in the questionnaire; however, if the answer was "no", the questionnaire form would be submitted directly. 

All information was kept private and was solely used for scientific research, and participation in this study was entirely voluntary and optional with informed consent provided to all participants on the first page before filling out the questionnaire. The ethical approval of the study was obtained before initiating the study. The ethical approval was obtained from the Biomedical Ethics Committee at Jazan University (reference No. REC- 43/10/219).

Statistical analysis

The collected data was first entered into a Microsoft Excel file and later transferred to SPSS version 23 for further analysis. The mean ± standard deviation (SD) was reported for continuous variables like age, while categorical variables like gender were described using frequencies and percentages. A chi-square test was used to compare categorical variables like gender and the presence of PFP. The p-value <0.05 was considered significant

## Results

A total of 1688 respondents filled out the questionnaire. After applying the exclusion criteria, 1558 participants were included in the final analysis of the study. About 663 (42.6%) were males and 895 (57.4%) were females. Of the total participants, 718 (46%) were within the age group of 18 to 25, 508 (32.6%) were within the age group of 26 to 35, and 332 (21.3%) were within the age group of 36 to 40. As for marital status, 814 (52.2%) of the participants were single, 687 (44.1%) were married, 44 (2.8%) were divorced, and 13 (0.8%) participants were widowed. Concerning the level of education, about 1007 (64.6%) of the participants held bachelor's degrees, 469 (30.1%) had a public education level, and 82 (5.3%) of the participants were postgraduates. Of the participants, 427 (27.4%) were from the northern region, 324 (20.8%) were from the western region, 310 (19.9%) were from the central region, 261 (16.8%) were from the southern region, and 236 (15.1%) were from the eastern region. 

About 472 of the participants had PFP which corresponds to a 30.3% overall prevalence of PFP among the current study participants. The prevalence of PFP among males was found to be 31.4% and the prevalence of PFP among females was found to be 29.5%. The overall prevalence of knee pain among study participants was found to be 13.2% as 205 of the participants reported knee pain. The prevalence of knee pain among males was found to be 14% and the prevalence of knee pain among females was found to be 12.3%. The baseline characteristics of the study respondents and the prevalence of PFP and knee pain are shown in Table 1.

**Table 1:** Baseline characteristics of the study respondents and prevalence of PFP and knee pain PFP: Patellofemoral pain

Variable		Total	Males	Female	P-value
		N (%)	N (%)	N (%)	
No. of subjects		1558 (100)	663 (42.6)	895 (57.4)	-
Age (years)	18 – 25	718 (46.1)	260 (39.2)	458 (51.2)	<0.001
	26 – 35	508 (32.6)	282 (42.5)	226 (25.3)	
	36 – 40	332 (21.3)	121 (18.3)	211 (23.6)	
Marital status	Single	814 (52.2)	355 (53.5)	459 (51.3)	0.047
	Married	687 (44.1)	294 (44.3)	393 (43.9)	
	Divorced	44 (2.8)	11 (1.7)	33 (3.7)	
	Widowed	13 (0.8)	3 (0.5)	10 (1.1)	
Educational level	Public Education	469 (30.1)	219 (33)	250 (27.9)	0.095
	Bachelor’s degree	1007 (64.6)	411 (62)	596 (66.6)	
	Postgraduate degree	82 (5.3)	33 (5)	49 (5.5)	
Residence	Northern	427 (27.4)	185 (27.9)	242 (27)	< 0.001
	Southern	261 (16.8)	105 (15.8)	156 (17.4)	
	Central	310 (19.9)	93 (14)	217 (24.2)	
	Eastern	236 (15.1)	98 (14.8)	138 (15.4)	
	Western	324 (20.8)	182 (27.5)	142 (15.9)	
PFP		472 (30.3)	208 (31.4)	264 (29.5)	0.426
Knee pain		205 (13.2)	95 (14.3)	110 (12.3)	0.239

The following demonstrates the univariate and multivariate logistic regression. The univariate analysis included the following variables: age, gender, marital status, and education. The following factors predicted higher rate of PFP: being 18 to 25 years old (p<0.001, odds ratio = 1), being 26 to 35 years old (p< 0.001, odds ratio = 1.627), being married (p = 0.005, odds ratio = 1.43). No factors predicted a lower rate of PFP. 

The multivariate analysis included the following variables: age, gender, and marital status. The following factors predicted higher rate of PFP: being 18 to 25 years old (p< 0.001, odds ratio = 1), being 26 to 35 years old (p = 0.001, odds ratio = 1.689). No factor predicted a lower rate of PFP. Logistic regression analysis to identify associated factors with the prevalence of PFP is shown in Table 2.

**Table 2:** Logistic regression analysis to identify associated factors with the prevalence of PFP PFP: Patellofemoral pain, OR: Odds ratio, CI: Confidence interval

Variable	With PFP N = 472	Without PFP N = 1086	P-value	Univariate logistic regression		Multivariate logistic regression	
				OR (95% CI)	P	OR (95% CI)	P
Age (years)					<0.001		<0.001
18 – 25	173 (36.7)	545 (50.2)	< 0.001	1		1	
26 – 35	173 (36.7)	335 (30.8)		1.627 (1.266 – 2.091)	<0.001	1.689 (1.251 – 2.280)	0.001
36 – 40	126 (26.7)	206 (19)		1.927 (1.456 – 2.549)	<0.001	2.127 (1.453 – 3.115)	<0.001
Gender							
Male	208 (44.1)	455 (41.9)	0.426	1		1	
Female	264 (55.9)	631 (58.1)		0.915 (0.736 – 1.138)	0.426	0.957 (0.763 – 1.200)	0.701
Marital status					0.006		0.417
Single	219 (46.4)	595 (54.8)	0.005	1		1	
Married	234 (49.6)	453 (41.7)		1.403 (1.125 – 1.751)	0.003	0.932 (0.689 – 1.261)	0.650
Divorced	12 (2.5)	32 (2.9)		1.019 (0.515 – 2.014)	0.957	0.709 (0.345 – 1.461)	0.352
Widowed	7 (1.5)	6 (0.6)		3.170 (1.054 – 9.535)	0.040	2.056 (0.654 – 6.464)	0.217
Education					0.291		0.117
Public education	133 (28.2)	336 (30.9)	0.291	1		1	
Bachelor’s degree	318 (67.4)	689 (63.4)		1.166 (0.916 – 1.483)	0.211	1.152 (0.900 – 1.475)	0.260
Postgraduate degree	21 (4.4)	61 (5.6)		0.870 (0.509 – 1.485)	0.609	0.695 (0.402 – 1.203)	0.194

## Discussion

Patellofemoral and knee pains constitute a considerable proportion of musculoskeletal abnormalities which could result in significant discomfort and a reduction in quality of life. Studying the prevalence of patellofemoral and knee pain will shed the light on the importance of having solid data about the exact disease burden on the community and thus more preventive and management options to be applicated [13]. The present study aimed to determine the prevalence of PFP and knee pain among the Saudi young adult population and to identify factors associated with the prevalence of PFP and knee pain. This is the first large-scale study examining the prevalence of PFP and knee pain in the general population of Saudi Arabia. 

The overall prevalence of PFP was found to be 30.3%. The prevalence of PFP among males was found to be 31.4% and the prevalence among females was found to be 29.5%. This prevalence is higher when compared to the prevalence reported in the study conducted by Xu et al. in which the overall prevalence was found to be 20.7% and prevalence of PFP among males was found to be 20.3% and PFP prevalence among females was found to be 21.2% [5]. The current study's overall prevalence was also found to be higher than which reported in the study carried out by Smith et al. in which the overall prevalence was found to be 22.7% [4]. This could be attributed to varying levels of activity and different ages of participants apart from genetic and environmental factors that could also play an important role, and therefore more studies are needed. Also, there may be variations in prevalence due to the personnel composition of the sample population and diagnostic techniques. 

The prevalence of knee pain among study participants was found to be 13.2% and this prevalence was found to be higher when compared to the other parallel study conducted by Nguyen et al. in which knee pain was found to be 8% [14], but this prevalence of knee pain in the current study was found to be lower than the prevalence in a study carried out by Chin et al. in which prevalence was found to be 21.1% [15].

The univariate analysis included the following variables: age, gender, marital status, and education. The following factors were found to be predicting a higher rate of PFP: being married, being 18 to 25 years old, and being 26 to 35 years old. These factors were found to be contradictory to the study conducted by Cook et al. which found no difference between age, gender, and PFP [16]. The multivariate analysis included the following variables: age, gender, and marital status. The following factors predicted a higher rate of PFP: being 18 to 25 years old and being 26 to 35 years old. Similar findings were reported in the congruent study conducted by Crossley et al. in which the prevalence of PFP was linked to ages less than 40 years old [17]. In the current study, PFP was found to be higher in females, but there is no significant difference. Patellofemoral pain is reported to occur more commonly in females than in males [18,19]. This could be due to differences in the biomechanics of the lower extremities compared to men. 

There are also emerging concerns regarding the long-term consequences of PFP, such as an increased incidence of patellofemoral osteoarthritis [20,21]. Such concerns regarding long-term impact imply that preventative strategies to lower the risk of PFP should be considered. Therefore, further studies that evaluate PFP and its associated risk factors are crucial to allow more feasible large-scale preventive action. The patellofemoral syndrome has a reasonably fair prognosis; however, if left untreated, it could severely limit a patient's mobility due to discomfort, or it can lead to patellofemoral osteoarthritis due to insufficient patella tracking [13]. Therefore, early detection and evidence-based treatment can decrease pain and enhance functional mobility, enabling patients to maintain an active lifestyle.

As with most cross-sectional studies, the limitations of our study were the inability to assess the incidence and to make a causal inference. The lack of a uniform definition of PFP is one of the main limitations. Also, convenience sampling brings out the possibility of under or over-representation of the population; therefore, we suggest conducting future studies using alternative study designs, such as a retrospective study for further accurate assessment of PFP and its related factors. We confined the age range between 18 to 40 years, which may influence the results to some extent. The prevalence may vary from the given age range in older or younger populations. Some children may be restricted from using mobile phones, and individuals older than 40 may develop osteoarthritis of the knee. Due to this, this study only included participants who were between the ages of 18 and 40. Awareness and knowledge about PFP and knee pains in terms of prevention and simple management procedures at home and when to visit the doctor should be raised. Population health education could be reached by encouraging the role of media and social community events about PFP and knee pains.

## Conclusions

The prevalence of PFP and knee pains in Saudi Arabia was found to be high compared to the parallel investigations. An age less than 40 years old was identified to be associated with a higher prevalence of PFP and knee pain when compared to other age groups. Higher-quality research employing different study designs is recommended to further investigate PFP and its related factors.
